# *In silico* docking and molecular dynamics for the discovery of inhibitors of enteric methane production in ruminants — A review

**DOI:** 10.5713/ab.24.0291

**Published:** 2024-08-26

**Authors:** Kamburawala Kankanamge Tharindu Namal Ranaweera, Myunggi Baik

**Affiliations:** 1 Department of Agricultural Biotechnology and Research Institute of Agriculture and Life Sciences, College of Agriculture and Life Sciences, Seoul National University, Seoul 08826, Korea; 2 Department of Animal Science, Faculty of Animal Science and Export Agriculture, Uva Wellassa University, Badulla 90000, Sri Lanka; 3 Institute of Green-Bio Science and Technology, Seoul National University, Pyeongchang 25354, Korea

**Keywords:** Computer Techniques, Enzyme Inhibitors, *In silico*, Methane, Methane Inhibitors, Ruminant

## Abstract

The increase in methane emissions, a major greenhouse gas, threatens human well-being and global ecosystems due to its contribution to global warming. Livestock, particularly ruminants, have been a major research topic in recent decades due to their methane production. Therefore, the objective of the current review was to comprehensively discuss the *in silico* techniques used to mitigate methane production from ruminants. The review covers the principles of *in silico* docking and molecular dynamics, which can be used to develop methanogenesis inhibitors. It also discusses specific methanogen enzymes as potential targets for inhibitor development. Furthermore, *in silico*-based methanogenesis inhibitor development studies have been reviewed with the authors’ opinions. The further use of *in silico*-based research techniques, including artificial intelligence-based systems, is encouraged to help reduce methane production from livestock more efficiently and cost-effectively.

## INTRODUCTION

### Global warming and greenhouse gas emissions

**Figure 1:**
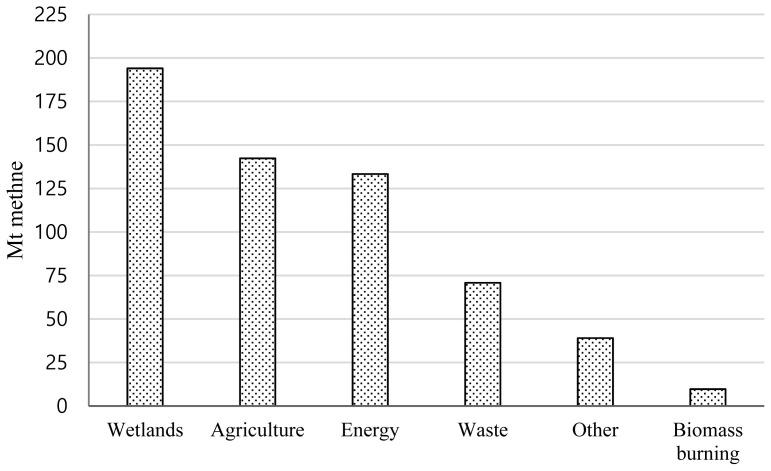
Sources of methane emissions in metric tons (Mt). Modified from the International Energy Agency [6], according to the Creative Commons License.

In July 2023, the world experienced the hottest month on record, surpassing the highest global mean surface air temperature in August 2016 [1]. The relationship between global temperature and the concentration of greenhouse gases (GHGs) in the atmosphere is well-established. Since the industrial revolution, the concentration of GHGs has steadily increased, disrupting the natural balance of GHG concentrations in the atmosphere and causing a significant impact on the earth’s temperature [2]. Notably, the agriculture sector, accounting for approximately 18.4% of emissions, is a significant contributor to anthropogenic GHG emissions [3]. Livestock and manure emissions contribute approximately 5.8% of total GHG emissions in the agricultural sector [4]. Methane is a potent GHG, with a global warming potential 28 times greater than carbon dioxide over a 100-year time frame. It also contributes to the formation of ground-level ozone, adversely affecting air quality [5]. Agricultural activities are the second-largest source of methane emissions after natural emissions from wetlands and peatlands (Figure 1) [6]. The livestock sector accounts for 30% of global anthropogenic methane emissions [7]. These emissions are projected to continue to rise due to the increasing global demand for milk and beef. Therefore, reducing methane emissions from the agricultural sector, particularly livestock, can significantly mitigate global warming.

### Strategies for mitigating livestock methane emissions

Methanogenic archaea, primarily colonized in the reticulorumen, play a significant role in enteric methane production. This methane is released into the environment through animal eructation. The conversion and removal of hydrogen to methane in the rumen is a crucial process for maintaining a negative redox potential, which is favorable for the growth and activity of other rumen microorganisms [8]. However, it is essential to note that this process also results in an inevitable loss of gross dietary energy in animal feed, ranging from approximately 2% to 12% [9]. Given the negative impact of rumen methane production, numerous studies have aimed to identify effective methods for inhibiting its production. The most prominent strategies employed in rumen methane mitigation over the past two decades have been those of feed formulation (forage quality, roughage to concentrate ratio, etc.), selection and breeding of low methane-producing animals, vaccines, methane reducing feed additives, and defaunation [10–14].

Many recent reviews have discussed these interventions in detail [9,15–17]. However, the interventions showed significant variations in their results and efficacy under both *in vitro* and *in vivo* conditions [18]. Chemical inhibitors of methanogenesis offer a direct and specific way to limit methane production. These inhibitors inactivate methanogen-specific enzymes and disable metabolic pathways to reduce methane production in the rumen without affecting other rumen microorganisms. Patra and Puchala [16] recently reviewed the mode of action of potential inhibitors of individual methanogenic enzymes in the methanogenesis pathway. In addition to the methanogenesis pathway enzymes, researchers have identified several other metabolic enzymes and proteins unique to methanogenic archaea. For more information on these enzymes and proteins, readers are encouraged to refer to work done by Attwood et al [19]. Most studies that identified unique proteins in methanogens employed whole genome analysis and comparative analysis of the gene sequences [20]. Inhibiting these unique, rate-limiting enzymes in methanogens can provide a more efficient and direct way to reduce rumen methane production without interfering with other beneficial rumen microorganisms. The discovery of these specific methanogenesis inhibitors necessitates an interdisciplinary approach, integrating expertise from diverse fields such as computational biology, enzymology, biochemistry, and animal sciences. However, to the best of the authors’ knowledge, no reviews have been published regarding the process of discovering specific methanogenesis inhibitors. Therefore, the objective of the present review is to address this gap and to encourage researchers to employ these diverse approaches in order to identify more efficient methanogenesis inhibitors.

### Methanogen-specific inhibitor development

Several effective methods exist for developing enzyme inhibitors for pathogenic microorganisms. These include *in silico* docking methods, high-throughput screening methods using enzyme assays, and high-throughput phenotypic screening methods using cell-based assays [15]. These methods can also be applied to methanogen-specific inhibitor discovery. While conventional inhibitor discovery and high-throughput screening methods have inherent advantages, they are expensive and time-consuming. Computer-aided drug discovery (CADD) methods or *in silico* methods are becoming more attractive for discovering methanogenesis inhibitors due to their scalability, time efficiency, and cost-effectiveness [21]. *In silico* docking is a computational technique employed in the fields of drug discovery and molecular biology. It predicts the orientation and binding affinity of a small molecule, such as inhibitors, within the protein’s active site. This information can facilitate the design of new inhibitors or the optimization of existing ones. Several recent reviews have discussed using CADD methods in human drug discovery projects [22–25]. However, a detailed review has yet to be conducted on the use of CADD methods in methanogen-specific inhibitor development. This review will comprehensively discuss studies using CADD methods to identify specific methanogenic inhibitors. The CADD methods used in the current context will be identified, and potential target methanogen enzymes for future inhibitor development studies will be reviewed. The following sections will briefly describe the *in silico* methods used in inhibitor discovery.

## TARGET MOLECULES FOR METHANOGENESIS INHIBITOR DISCOVERY

Archaea were identified as a separate domain in the 1970s by American microbiologist Carl Richard Woese and his colleagues [26]. Archaea are further classified into four kingdoms: *Euryarchaeota*, *Crenarchaeota*, *Korarchaeota*, and *Nanoarchaeota*. Methanogens are included in the kingdom *Euryarchaeota* and consist of mainly five orders: *Methanobacteriales*, *Methanococcales*, *Methanomicrobiales*, *Methanosarcinales*, and *Thermoplasmatales*. The distribution of methanogen species in the rumen and their function have been extensively described in the scientific literature, as demonstrated by the works of Patra et al [18]. Methanogens have unique physiological characteristics, including metabolic pathways distinct from other rumen microorganisms. This is beneficial because inhibiting rumen methanogenesis should not disrupt other rumen microorganisms (bacteria, fungi, and protozoa) involved in proper digestion. However, inhibiting ruminal methane emissions may result in changes to ruminal hydrogen partial pressures and fermentation parameters, which can indirectly affect the composition of the ruminal microbiome [8]. Studies have found several methanogen-specific enzymes that could be targeted for inhibitor development [15]. Furthermore, genome sequencing studies have identified novel unique genes and their expressed proteins that could be methanogen-specific targets [20]. This section of the review aims to describe enzyme targets for methanogenesis inhibitor development and *in silico* inhibitor discovery.

### Enzymes related to the methanogenesis pathway

**Figure 2:**
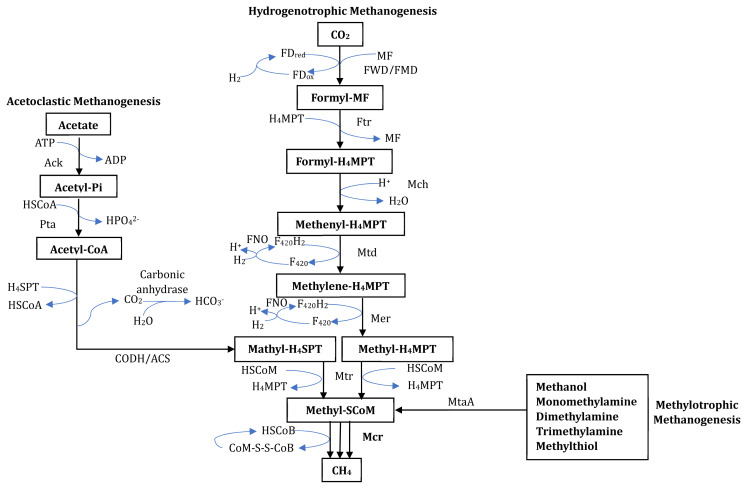
Enzymes involved in the methanogenesis pathways observed in archaeal methanogens. Ack, acetate kinase; ATP, adenosine triphosphate; ADP, adenosine diphosphate; CODH/ACS, carbon monoxide dehydrogenase/acetyl-CoA synthase; FDred/ox, ferredoxin (reduced/oxidized); FMD, Mo-containing formylmethanofuran dehydrogenase; FNO, coenzyme F420H2:NADP+ oxidoreductase; Ftr, formylmethanofuran-tetrahydro methanopterin; FWD, W-containing formylmethanofuran dehydrogenase; H4MPT, tetrahydromethanopterin; H4SPT, tetrahydrosarcinapterin; HSCoA, coenzyme A; HSCoB, coenzyme B; HSCoM, coenzyme M; Mch, methenyl-tetrahydromethanopterin cyclohydrolase; Mcr, methyl coenzyme M reductase; Mer, 5,10-methylene tetrahydromethanopterin reductase; MF, methanofuran; MtaA, coenzyme M methyltransferase; Mtd, methylene tetrahydromethanopterin dehydrogenase; Mtr, methyl-H4MPT coenzyme M methyltransferase; Pta, phosphate acetyltransferase. Modified from Lambie et al [27], according to the Creative Commons License.

Methanogenesis is the energy-producing pathway in methanogenic archaea (Figure 2) [27]. The details of this pathway have been extensively described elsewhere [28]. Several unique enzymes are currently targeted for inhibitor development in this pathway.

#### Methyl coenzyme M reductase

Methyl coenzyme M reductase (MCR) (EC 2.8.4.1) is a central enzyme in the methanogenesis pathway due to its participation in reducing methyl coenzyme M with coenzyme B to produce methane and coenzyme M-coenzyme B heterodisulfide [29]. MCR is one of the most well-studied enzymes present in the methanogenesis pathway. MCR has three isoenzymes: MCRI, MCRII, and MCRIII. MCR exists as a dimer of heterotrimers (*α*2*β*2*γ*2), and the nickel tetrahydrocorphinoid coenzyme (F430) is located at the enzyme’s active site. The enzyme is in its active state with the Ni ion in a 1+ oxidation state. Several studies have characterized MCR activity and its structural properties [30,31]. Although methane production could start with different substrates (according to different methanogen species) such as CO_2_, methanol, formate, acetate, and methylamine, all three methanogenesis pathways merge in the last two steps of methanogenesis. Since MCR is involved in the last step of the pathway, it acts as a common target for all methanogen species, making it a valuable target enzyme for inhibiting methanogenesis in ruminants.

**Figure 3:**
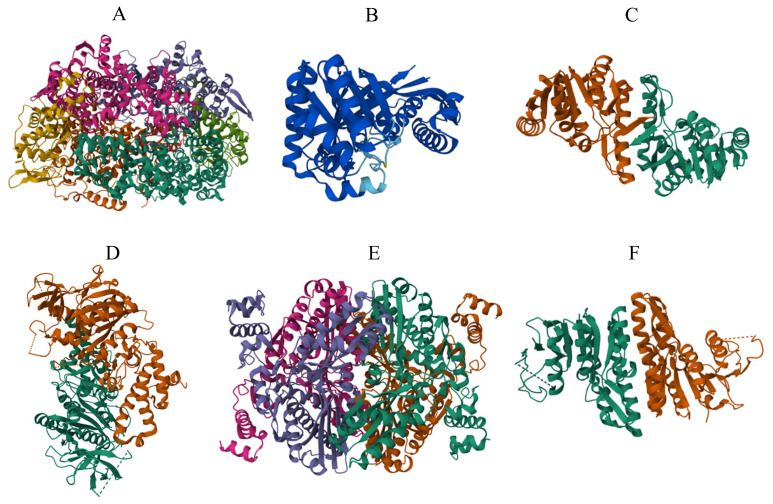
The 3D ribbon structure of target molecules for methanogenesis inhibitor discovery. (A) Methyl coenzyme M reductase (*Methanothermobacter thermautotrophicus*). Image from the RCSB PDB (RCSB.org) of PDB ID 1HBN [29,32,33]. (B) Predicted structure of 4-(*β*-D-ribofuranosyl) aminobenzene-5’-phosphate synthase (AlphaFoldDB: Q58822, EC 2.4.2.54, [41]). (C) Coenzyme F420H2:NADP+ oxidoreductase (FNO) from *Archaeoglobus fulgidus*. Image from the RCSB PDB (RCSB.org) of PDB ID 1JAY [32,45]. (D) Subunit B of the A1A0 ATP synthase from *Methanosarcina mazei*. Image from the RCSB PDB (RCSB.org) of PDB ID 3DSR [32,50]. (E) HMG-CoA reductase from *Methanothermococcus thermolithotrophicus*. Image from the RCSB PDB (RCSB.org) of PDB ID 6HR8 [32,56]. (F) Isopentenyl phosphate kinase from *Thermoplasma acidophilum* Image from the RCSB PDB (RCSB.org) of PDB ID 3LKK [32,58].

The high-resolution X-ray crystal structures of MCR could be used for *in silico* inhibitor discovery studies (Figure 3A) [29,32,33]. MCR inhibitors were first discovered in the 1970s. At that time, researchers discovered that the compound 2-bromoethanesulfonate inhibited methanogenesis from acetate in *Methanosarcina* species. Subsequently, other compounds, such as 2-chloroethanesulfonate and 2-mercaptoethanesulfonate, have also been identified as inhibitors of MCR [34]. These compounds are structural analogs of the natural substrate of MCR, methyl-coenzyme M. By binding to the enzyme’s active site, they block the normal substrate from binding [35]. Discovery of these inhibitors typically involved biochemical assays that test the ability of potential inhibitory compounds to reduce the activity of MCR. These inhibitors are valuable tools for studying methanogenesis and have potential applications in mitigating ruminant methane emissions.

Furthermore, researchers have recently used computational methods to predict potential MCR inhibitors. Large datasets can be analyzed to screen for inhibitors and expedite discovery efficiently. An excellent example of an inhibitor discovered through *in silico* methods is 3-nitrooxypropanol (3-NOP), which will be discussed in the following sections of this review.

#### 4-(β-D-ribofuranosyl) aminobenzene-5’-phosphate synthase

The enzyme 4-(*β*-D-ribofuranosyl) aminobenzene-5’-phosphate (RFA-P) synthase (EC 2.4.2.54) catalyzes the first step (shown below) in tetrahydromethnopterin (H_4_MPT) biosynthesis.


$$\begin{array}{l} 4\text{-hydoxbenzoate}+5\text{-phospho}-\alpha \text{-D-ribose }1\text{-diphosphate}+{\text{H}}^{+}\to \\ 4-(\beta \text{-D-ribofuranosyl})\text{hydroxybenzene }5^{\prime }\text{-phosphate}+{\text{CO}}_{2}+\text{diphosphate} \end{array}$$

H_4_MPT is a coenzyme in the methanogenesis pathway, carrying the C1 group as it is reduced to the methyl level before transferring to the coenzyme M [36]. Hence, inhibiting the synthesis of H_4_MPT by inhibiting RFA-P synthase may eradicate methane production. This enzyme polypeptide has a molecular weight of 36.198 kD and is categorized as a transferase [37]. Dumitru and Ragsdale [38] did an initial study on the mechanism of RFA-P synthase, and a precise Bi-Ter mechanism was suggested. After identifying this as a potential target for methane mitigation, an inhibitory study was conducted by Dumitru et al [39] using an enzyme assay method combined with inhibitors. The researchers suggested analogs of p-aminobenzoate as potential inhibitors. Specifically, the p-aminobenzoate derivatives containing isopropyl, n-propyl, and isobutyl nitrogen substituents were identified as potent inhibitors of RFA-P synthase. Further, Miner et al [40] patented the potential variants of the RFA-P synthase inhibitors. An enzyme structure is required for development of an *in silico* inhibitor. Researchers have not yet elucidated the RFA-P synthase enzyme structure using X-ray crystallography, but a predicted structure generated by the AlphaFold program could be obtained in the UniProt database under the EC number 2.4.2.54 (Figure 3B) [41]. This predicted structure could be used for *in silico* inhibitor discovery experiments combined with post-modeling refinements.

#### Coenzyme F420H2:NADP^+^ oxidoreductase

The F420 cofactor plays a vital role in the methanogenesis pathway by serving as a hydride carrier and aiding in reducing carbon dioxide to methane. It reduces methenyl tetrahydromethanopterin to methylene tetrahydromethanopterin, an important step in the methanogenesis pathway. Additionally, it is involved in reducing methylene tetrahydromethanopterin to methyl tetrahydromethanopterin, another crucial step in the pathway. Therefore, the F420 cofactor plays a central role in methanogenesis, facilitating critical redox reactions that would otherwise be thermodynamically unfavorable [42]. The coenzyme F420H2:NADP^+^ oxidoreductase (FNO) enzyme facilitates the reduction of NADP^+^ to NADPH by using F420H_2_ as the electron donor. In the other direction, it catalyzes the oxidation of NADPH to NADP^+^ with the concomitant reduction of F420 [43].


$${\text{NADP}}^{+}+\text{reduced cofactor }{\text{F}}_{420}↔\text{NADPH}+\text{oxidized cofactor }{\text{F}}_{420}+{\text{H}}^{+}$$

This reaction is important for maintaining methanogenesis, as it reduces CO_2_ to methane. Hence, inhibition of FNO may interrupt the oxidation and reduction mechanism of the F420 cofactor and ultimately reduce methane production [44]. The FNO enzyme (EC 1.5.1.40) has a molecular weight of 21.0 kD and is considered an oxidoreductase [37]. The 3D structure of FNO (discovered by the X-ray diffraction method) is available through the RCSD database (ID: 1JAY) (Figure 3C) [32,45]. However, the structure was developed based on the archaeal species *Archaeoglobus fulgidus*, and a structure specific to rumen methanogens has not yet been obtained [45]. Nonetheless, homology modeling may generate a 3D structure of the rumen-specific methanogen FNO based on the available structure and amino acid sequences, as demonstrated by Cuccioloni et al [44]. Homology modeling is a method employed in structural biology to predict the three-dimensional structure of a protein. If the structure of a similar protein is known, it can be utilized to model the structure of the target protein using its amino acid sequence. The process involves sequence alignment, model building, and model refinement. It is a valuable tool in the absence of experimental data, such as when crystallography or NMR structures are not available. Considering the relatively limited number of *in silico* studies conducted on this enzyme, it is possible that future research could lead to the discovery of novel inhibitors.

Recently, research groups have focused on other critical enzymes and proteins outside the methanogenesis pathway in rumen archaea. The development of more efficient methanogenesis inhibitors is still needed. More target enzymes to methanogens could be used to develop inhibitors of methane production. In the present review, the authors have aimed to provide insight into some of these enzymes so that future research can focus on the development of more diverse and effective inhibitors.

### A1A0 adenosine triphosphate synthase

Adenosine triphosphate (ATP) synthases are proteins that catalyze the formation of the energy storage molecule ATP from adenosine diphosphate and inorganic phosphate. There are four main classes of ATP synthases based on their functional differences: F (phosphorylation factor), V (vacuole), A (archaea), and E (extracellular) [46]. The A1A0 ATP synthase in archaea is a membrane-bound enzyme comprised of subunits A through K. It shares structural and functional similarities with V-ATPase and F-ATPase, respectively [47]. The structural differences among ATP synthase types have made them potential enzyme targets for treating human diseases and inhibiting archaea [48]. Furthermore, the complex structure of ATP synthases makes them vulnerable to various inhibitors [46]. The structural features of A1A0 ATP synthase were discussed in detail by Schäfer et al [47] and Vonck et al [49]. Additionally, Aung et al [48] proposed a method for discovering inhibitors of archaeal ATP synthase using a high-throughput screening assay. However, to use *in silico* methods, it is imperative to acquire 3D structures of this enzyme. Only the structure of subunit B of A1A0 ATP synthase is known from X-ray diffraction studies and is accessible in the RCSB database (Figure 3D) (RCSB ID: 3DSR) [32,50]. Homology modeling methods could be employed to anticipate additional structures for further *in silico* research.

### The mevalonate pathway in archaea

**Figure 4:**
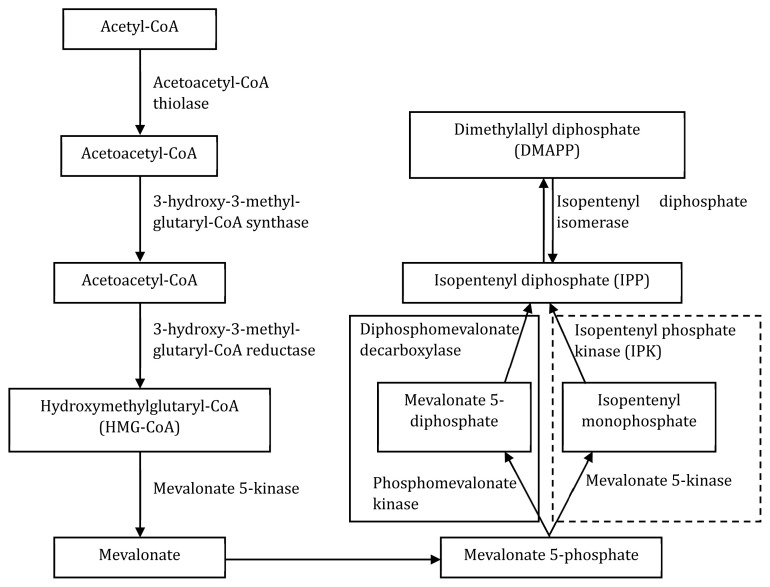
Mevalonate pathways found in eukaryotes (pathway through solid line box) and archaea (pathway through dotted line box). Modified from Johnson et al [52], according to the Creative Commons License.

In eukaryotic and bacterial cell membranes, fatty acid chains are linked via an ester bond to sn-glycerol-3-phosphate. However, the isoprenoid chains are bound with an sn-glycerol-1-phosphate in archaeal cell membranes via an ether link [51]. Archaea use isopentenyl diphosphate and dimethylallyl diphosphate as precursors to assemble their isoprenoid units. These building blocks are synthesized via the mevalonate pathway, also present in Bacteria and Eukarya. However, the last two enzymes in the archaeal mevalonate pathway differ from the Bacteria and Eukarya pathways (Figure 4) [51,52]. Inhibition of the mevalonate pathway in archaeal cells may disrupt the cell membrane, which could ultimately lead to cell death [53]. As a result, targeting the archaeal mevalonate pathway could be a potential method for mitigating methanogens in the rumen.

#### Hydroxymethylglutaryl – CoA reductase

Hydroxymethylglutaryl – CoA reductase (HMG-CoA reductase) is involved in converting hydroxymethylglutaryl-CoA to mevalonate, the rate-limiting step of the mevalonate pathway [54]. Several *in vitro* studies have shown that the statins mevastatin and lovastatin are promising inhibitors of HMG-CoA reductase under *in vitro* conditions and reduce methane production by inhibiting methanogens [53]. However, it is important to consider the cost implications of using statins as a feed additive to reduce methane in ruminants [55]. Exploring alternative and cost-effective inhibitors that specifically target this enzyme may be beneficial to address methane reduction. The 3D structure of HMG-CoA reductase (generated by the X-ray diffraction method) from the methanogen *Methanothermococcus thermolithotrophicus* is available in the RCSB database (RCDB ID: 6HR8) (Figure 3E) [32,56]. Currently, there are no available 3D structures for this particular rumen methanogen enzyme. As a result, methods such as homology modeling could generate appropriate structures for *in silico* docking and dynamics studies.

#### Isopentenyl phosphate kinase

The mevalonate pathway in most archaeal species (including methanogens) differs from the classical mevalonate pathway in eukaryotes. In the classical mevalonate pathway, the diphosphorylation of MVA-5-phosphate occurs before the decarboxylation step. However, in most archaea, the phosphorylation step occurs after decarboxylation by a unique enzyme called isopentenyl phosphate kinase (IPK) [57]. The alternate mevalonate pathway presents an opportunity to develop inhibitors targeting IPK. These inhibitors could disrupt archaeal cell membrane synthesis, inhibiting cell growth and viability. IPK belongs to the amino acid kinase family, and its structure and function have been extensively studied. The structure of IPK is well-defined, and the 3D structures generated via X-ray diffraction could be obtained in the RCSB database (RCSB ID: 3LKK) (Figure 3F) [32,58]. Although the enzyme structure for IPK was not analyzed in rumen methanogens, the amino acid sequence of IPK in rumen methanogens (specifically *Methanobrevibacter ruminantium*) shows higher sequence similarities with available structures from archaea (such as *Thermoplasma acidophilum*). Hence, currently available 3D structures of IPK (i.e., RCSB ID: 3LKK) could be used for *in silico* experiments [58,59].

The enzyme targets discussed in this section could be used for future *in silico* research to discover new, more efficient rumen methanogenesis inhibitors. However, as discussed above, high-resolution 3D structures of enzymes are necessary for better *in silico* work. Therefore, developing unique methanogen enzyme 3D structures using X-ray crystallography and NMR spectroscopy is another avenue for future research. The following sections will explore how these enzyme targets are used in *in silico* inhibitor discovery.

## *IN SILICO* SCREENING STUDIES FOR METHANOGENESIS INHIBITOR DISCOVERY

### *In silico* screening

*In silico* refers to the use of computer-based methods to simulate experiments. *In silico* pharmacology uses computational approaches to model biological processes, address therapeutic interventions, and simulate biological activities. These interventions are expected to predict, discover, and improve therapeutics [60]. In the drug discovery process for therapeutic purposes, virtual screening is a computational technique that can be used to search for potential drug candidates in virtual libraries containing many compounds [61]. Virtual screening uses *in silico* docking techniques to find the complementarity between a protein target and ligand, identifying possible compounds that could modulate protein activity [23].

### In silico docking

Ligand-based virtual screening and structure-based virtual screening are the two major methods used in CADD [62]. Protein structure information is typically obtained from experimental techniques such as X-ray crystallography or nuclear magnetic resonance and is necessary for a successful structure-based virtual screening [63]. The structures identified by these techniques can be accessed through databases such as BRENDA ( www.brenda-enzymes.org ), UniProt ( www.uniprot.org ), and RCSB PDB ( www.rcsb.org ). The initial step of structure-based virtual screening is identifying the target protein or the enzyme.

The suitability of the binding site in the target molecule for drug development can be assessed using bioinformatic tools such as ProteinPlus ( https://proteins.plus ). These tools identify the most appropriate binding sites based on the 3D structure of the target molecule, including geometric and energetic information. The probability that a drug can target the identified cavities has been evaluated using volume, hydrophobicity, and enclosure-like properties as inputs in various machine-learning models [64]. The 3D structure of the target molecule is an essential aspect of structure-based virtual screening. However, in cases where the 3D structure is unavailable, homology modeling may be useful for predicting structures. A detailed description of homology modeling is available in the review by Muhammed and Aki-Yalcin [65]. X-ray crystallography can also identify ligand binding sites, where the macro molecule (protein) and micro molecule (ligand) are co-crystallized. Furthermore, when obtaining the protein structures, the ligand-bound form of the enzyme (holo conformation) structure is more suitable than the ligand-unbound (apo conformation) structure in docking studies [66].

After obtaining the high-resolution protein structures, it may be necessary to modify them before use, as the original form may not be suitable for the intended purpose. These steps include verifying mutations in the protein structure, eliminating buffer components and cofactors (unless they are involved in the ligand interactions), removing water molecules (depending on the docking program used), stabilizing the charges, replacing missing atoms/residues, and adding hydrogen atoms. After the protein has been prepared, ligand libraries are prepared for docking.

### Ligand libraries

Selecting compounds from a collection of available databases to discover chemicals for a specific purpose (e.g., enzyme inhibition) is called library preparation [67]. The preparation of the ligand database is an essential factor in the success of the docking program [25]. The selection of ligands for docking will be based on the specific project objectives. The ligands can be retrieved from different databases, or the researcher can design ligands using different programs (i.e., ChemSketch available at https://www.acdlabs.com/ ). A few examples of the ligand databases available with ligand structure files are shown in Table 1 [68–75]. For a more extensive overview of natural product-based ligand databases, readers may refer to the work by Sorokina and Steinbeck [76]. Researchers can apply filters to the databases containing millions of molecules to optimize computational resources and time. Filters used in inhibitor discovery may include molecular weight, net charge, solubility, polar surface area, pharmacophores, commercial availability, absorption, distribution, metabolism, excretion, accessibility of synthetic compounds, and toxicity [61]. Incorporating Lipinski’s rule of five and searching for similarities to known active ligands are two additional filters [25]. When preparing the ligand library, it is important to consider the number of ligands and the relevance of the selected ligands by using information such as known active ligands (e.g., 3-NOP for MCR enzyme) or binding pocket information. Without information on the active ligands, diverse compounds could be used as a potential docking library [61]. Furthermore, verifying the precision of ligand geometries in the source database may be beneficial, as docking programs do not necessarily optimize ligands for their bonds and lengths.

Before docking, selected ligands should be prepared through charge assignment, geometry optimization, and conformation generation. Specifying the active site-directed docking site is possible once the ligands and target protein have been prepared. If the active site was not identified, blind docking could be performed [77]. Docking is conducted with the prepared ligands and target protein using docking software. A list of docking software used for *in silico* docking is shown in Table 2 [78–87]. After the ligands are docked against the enzyme or protein, the ligand-protein interactions are assessed, and a scoring function is used to select the best ligand-protein complex or complexes [88]. The docking algorithm may vary depending on the docking program used. The algorithms can be classified into three categories: shape matching, systematic search, and stochastic search algorithms [89]. Post-docking analysis is conducted to select the most promising ligands. The binding affinities are evaluated, and the ligands are ranked according to the best binding affinity. Further, the interactions such as hydrogen bonds, hydrophobic interactions, and electrostatic interactions between the ligand and protein are assessed [77].

**Figure 5:**
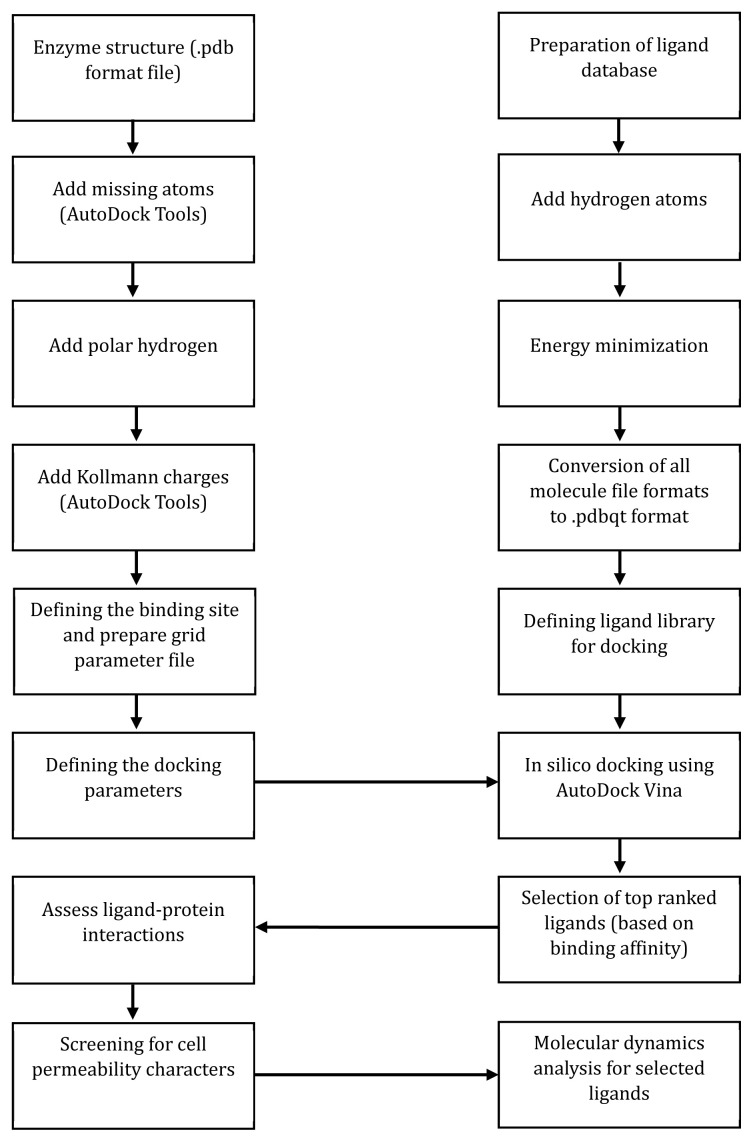
Summary of the *in silico* procedure followed in the authors’ laboratory for finding novel methanogenesis inhibitors.

While docking simulations can provide valuable information on the affinity of ligand-protein binding and the conformation of the ligand when binding to the active site, they may not necessarily provide a complete picture of the stability of the protein-ligand complex over time. Moreover, ligand-protein interactions can be influenced by protein flexibility and the presence of water molecules in the medium, which may not be considered in the docking process [22]. Another approach in the *in silico* inhibitor discovery process could be introduced as a potential solution to these issues, such as molecular dynamics studies. The docking procedure used in the authors’ laboratory for methanogenesis inhibitor discovery using Autodock Tools software and AutoDock Vina is summarized in Figure 5 [90,91].

#### Molecular dynamics

Performing molecular dynamics (MD) simulations after docking is common practice in *in silico* inhibitor discovery. MD is a computational simulation technique that relies on molecular mechanics. It allows the movement of individual particles within model systems to be investigated over time [92].

In addition to docking simulations, MD considers various factors such as solvent interactions, conformational sampling, and receptor clustering to provide a more comprehensive understanding of the conformational dynamics and stability of the ligand-protein complex. However, docking simulations alone may not fully consider solvent effects. These considerations contribute to a more comprehensive understanding of the ligand-protein complex’s conformational dynamics and, finally, stability of the complex [21]. Several software packages for performing MD exist, including Gromacs [93], AMBER [94], NAMD [95], CHARMM [96], and Desmond [97]. These widely used MD programs offer comparable features and may enhance performance by harnessing the computational capabilities and speed of graphics processing units [24].

Classical MD simulations involve Newton’s equations of motion to calculate particle paths based on an initial configuration. The total force acting on every particle in the system arises from interactions with other particles, as modeled by the forcefield [92]. The acceleration of a particle in the system is obtained by dividing the aforementioned force. Combining this acceleration with the particle’s previous position and velocity determines its new position after a short time interval [98]. These simulations reveal intricate details, including the trajectory of a ligand entering the binding pocket and the formation and evolution of protein-ligand intermediate states. Ultimately, MD provides an atomic-resolution explanation for the binding mechanism [99]. The preparation and execution of simulations in MD using different software packages may involve system-specific steps. However, a basic standard guideline can be provided.

**Figure 6:**
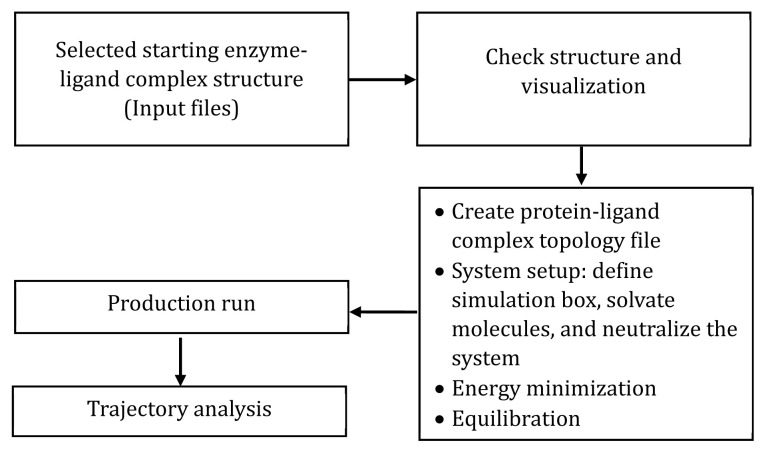
General steps of molecular dynamics simulation of an enzyme-ligand complex.

The major steps in MD simulation include preparing input structures and the system, running the production simulation, and analyzing the results obtained from the production run (Figure 6). The starting structures obtained from docking are used in the simulation, including the ligand-protein complex selected for its high interaction and affinity. Thoroughly checking the chemical structures for any missing atoms and modified ligands compatible with the forcefield parameters is recommended. Next, it is advisable to prepare the topology files, defining periodic boundary conditions, performing energy minimization of structures, adding solvent and ions, and setting temperature and pressure parameters to obtain the equilibrated solvated system. Factors such as the time duration for the simulation, the number of frames needed, and the necessity for storing velocities should be determined before running the production simulation.

**Figure 7:**
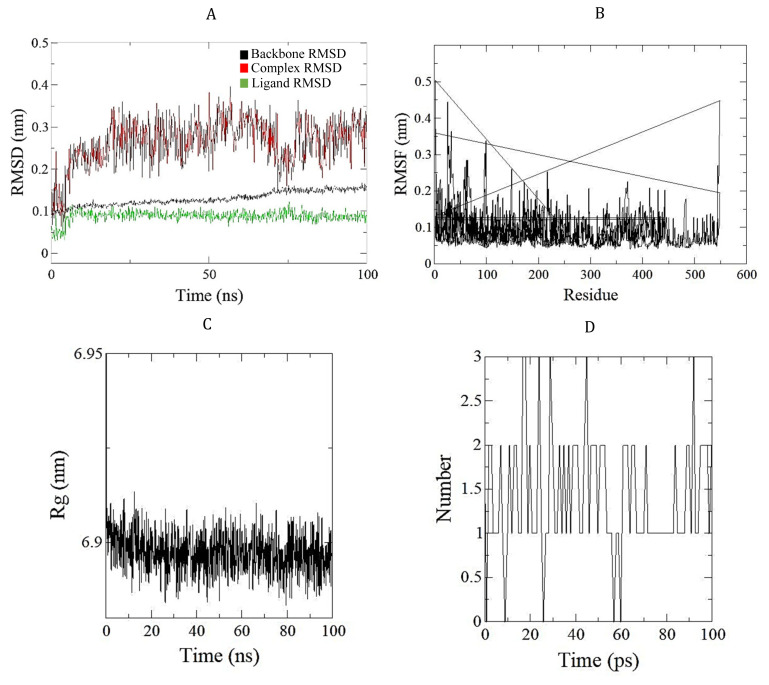
Example of the graph spectra under common parameters. (A) Root mean square deviations (RMSD). (B) Root mean square fluctuations (RMSF). (C) Radius of gyration (Rg). (D) Hydrogen-bonds generated by molecular dynamics simulation process. Graphs were derived from an MD simulation performed at the authors’ laboratory for an MCR enzyme-ligand complex. MD, molecular dynamics; MCR, Methyl coenzyme M reductase.

Following the production simulation, trajectory graphs will be presented, showcasing the results. While the results obtained from MD simulations may vary depending on the project’s objective, as MD is used in various fields, this review will specifically focus on commonly used results in ligand-protein simulations, particularly emphasizing those valuable in the inhibitor discovery process. The main objective of the results analysis is to identify parameters that indicate a stable interaction between the ligand and protein during the simulation period. The most common computed parameters include root mean square deviations (RMSD), root mean square fluctuations (RMSF), radius of gyration (Rg), and H-bond between ligand and protein [22]. An example of RMSD, RMSF, Rg, and H-bond graphs generated in the authors’ laboratory for an MCR enzyme-ligand complex is shown in Figure 7.

The RMSD quantifies the difference between a protein’s backbone in its initial structural conformation and its final position. By analyzing the deviations that arise during the simulation, one can evaluate the protein’s stability in relation to its conformation. Smaller deviations indicate a more stable protein structure [100]. Ligand RMSD can provide insights into the stability of the ligand relative to the protein and conformational changes over time [101]. The RMSD graph in Figure 7A shows that the enzyme backbone RMSD remains stable throughout the simulation with minor fluctuations, generally below 0.2 nm. This indicates that the overall structure of the protein backbone remained consistent throughout the simulation. Additionally, the red line represents the RMSD of the ligand in reference to the enzyme. The observed line exhibits fluctuations ranging from approximately 0.2 to 0.5 nm, indicating a dynamic interaction between the ligand and the enzyme. The RMSD of the ligand is represented by the green line, which is comparatively more stable than the red line but still exhibits some variability. The graph indicates that the ligand’s position relative to the enzyme had changed, but its conformation remained stable. The graph highlights the stable nature of the protein backbone, the dynamic interaction between the enzyme and the ligand, and the relative conformational stability of the ligand.

The RMSF graph can indicate the stable conformation of the selected set of protein residues. Areas of the protein that deviate significantly from its mean structure may indicate the presence of active sites resulting from ligand interactions with the binding site [102]. Figure 7B illustrates the RMSF values in nanometers (nm) for each residue in an enzyme-ligand complex. Higher RMSF values indicate greater flexibility or mobility of the residue. The x-axis represents the residue number, while the y-axis represents the magnitude of the fluctuation of each residue during the simulation. The graph reveals that specific residues exhibit higher RMSF values. The regions of high flexibility in the enzyme may play a crucial role in its function, such as binding to the ligand or undergoing conformational changes. Conversely, residues with lower RMSF values are more rigid or stable. This analysis provides information on the dynamic behavior of the enzyme-ligand complex and can help identify key residues critical for the enzyme’s function or interaction with the ligand.

The Rg indicates the distribution of residue atoms around the axis of a protein. The Rg of a protein will be affected when a ligand is bound to the active site. This helps assess the binding patterns of ligand-protein complexes [56]. Figure 7C shows that the Rg values fluctuate between approximately 6.9 nm and 6.95 nm throughout the simulation, indicating that the enzyme-ligand complex is undergoing conformational changes during the simulation, with the size of the complex expanding and contracting slightly over time. These changes in the radius of gyration could be attributed to various factors, such as the binding and unbinding of the ligand, changes in the conformation of the enzyme, or thermal fluctuations.

The stability of the complex can be inferred from the hydrophobic interactions, including the H bonding pattern between the protein residues and ligands. Higher interactions indicate a greater modulation ability of the ligand towards the protein. Conversely, a decreased number of H bonds may lead to deviations in the ligand-protein complex and a decrease in the stability of ligand binding [22]. Figure 7D shows that the number of hydrogen bonds fluctuates between approximately 0 and 3 during the simulation. These fluctuations indicate that the enzyme-ligand complex undergoes dynamic changes over time. The formation and breaking of hydrogen bonds may be attributed to ligand binding and unbinding, changes in enzyme conformation, or thermal fluctuations. The complex is stable if the number of hydrogen bonds remains relatively constant over time. However, this graph shows significant fluctuations in the number of hydrogen bonds, which may indicate that the complex is not highly stable. This could affect the enzyme’s function or the ligand’s efficacy as a drug. Additionally, stability of the complex can be influenced by various factors, including van der Waals interactions, electrostatic interactions, and hydrophobic effects. These analyses may assist in selecting stable protein-ligand structures and appropriate enzyme-modulating compounds for further evaluation in *in vitro* and *in vivo* systems. Collectively, *in silico* docking aids to narrow down a vast scale of chemical libraries into several hundreds of candidates screened according to best binding scores and binding poses. Additional screening steps could be used to further screen the compounds such as, the compound solubility, easiness and cost of synthesis, and toxicology of the compounds. Screening using these steps will result in selection of few candidates which could be tested with molecular dynamics analysis as discussed in above section. The most stable compounds selected from molecular dynamics analysis can then be subjected to *in vitro* testing. Following the *in silico* selection, the potential candidates should be evaluated for their inhibitory activity in *in vitro* and *in vivo* experiments. This involves determining the minimum concentration of inhibitor compound that mitigates methane production. Moreover, the impact of the inhibitors on rumen fermentation parameters (e.g. dry matter digestibility, ammonia and volatile fatty acid production, and pH) should be evaluated to ensure the normal rumen digestibility of the feed. Rumen microbial profiles may be analyzed to confirm the specific inhibitory effect on methanogens.

## DISCOVERY OF METHANOGEN-SPECIFIC INHIBITORS THROUGH *IN SILICO* ANALYSIS

Several studies have aimed to find methanogenesis inhibitors using various software and methods for *in silico* screening. The most successful methanogenesis inhibitor, 3-NOP, was discovered using a molecular docking program. In this study, Duin et al [33] targeted the MCR enzyme (PDB ID: 1HBN) in the methanogenesis pathway and prepared the ligand libraries for docking using pharmacophore modeling. Preparing a ligand library using pharmacophore modeling involves generating a set of ligands with diverse chemical structures and properties based on a pharmacophore model created from the enzyme binding site. The model was developed using LigandScout 3.1, a commercial software from Inte:Ligand GmbH. The selected ligands were uncharged and able to diffuse into cells. Molecular docking was conducted using Glide software. The accuracy of the findings was confirmed through enzyme assays and *in vitro* microbial inhibitory assays under laboratory conditions.

Phytochemicals are commonly used in inhibitor discovery, as demonstrated by Arokiyaraj et al [103] who employed 35 phytochemical compounds found in the rhubarb plant for *in silico* inhibitor discovery. These phytochemicals were selected based on a previous study by Kim et al [104], who suggested that rhubarb may reduce methane production under *in vitro* and *in vivo* conditions. Therefore, the study conducted by Arokiyaraj et al [103] can be considered a conformational study to support the results of the *in vitro* and *in vivo* studies. The ligands were prepared using Open Babel in the PyRx 0.8 software package, and docking was performed using AutoDock Vina in PyRx 0.8 with MCR (PDB ID: 1MRO) as the target enzyme. To confirm their effectiveness, the researchers re-docked the selected binding ligands using a different software (AutoDock Tools). The study used binding affinity (−6.92 kcal/mol to −5.61 kcal/mol) to select the top three ligands. Ligands were assessed for their adsorption, distribution, metabolism, and excretion (ADME) properties. According to Lipinski’s rule of five discussed in the above sections, 9,10-anthracene-dione, 1,8-dihydroxy-3-methyl was selected as the best candidate ligand to potentially inhibit the MCR. The ligand-protein interaction analysis between the aforementioned ligand and MCR indicated a strong hydrogen bonding pattern. Hydrogen bonds are considered the most important physical interaction in biomolecule systems in aqueous solutions and play a crucial role in ligand-protein interactions. Hydrogen bonds are also one of the factors for molecular recognition [105]. However, the MD analysis did not confirm this study’s results. Additionally, an *in vitro* or enzymatic assay was not performed using the purified chemical, which may have proven its activity under experimental wet chemistry conditions.

Dinakarkumar et al [106] conducted a study using 168 phytochemicals that targeted the MCR enzyme (PDB ID: 5A8K). The researchers used Lipinski’s rule of five to sort the ligands at the beginning of the study. This screening selected 51 phytochemicals for *in silico* docking. After evaluating the binding affinities and interactions (25 ligands were selected), the researchers evaluated the top three ligands with MD analysis. Through the RMSD, RMSF, and interaction plots, a compound named (3R,3aS,6R,6aR) - 3 - (2H-1,3-benzodioxol-4-yl) - 6 - (2H-1,3-benzodioxol-5-yl) - hexahydrofuro [3,4-c] furan-1-one showed the best binding stability during the 20 ns simulation time. However, it may be beneficial to consider an *in vitro* assessment of the selected compounds in future studies, as this was not included in the study. Docking studies have also been conducted to discover MCR inhibitors using phytochemicals from *Moringa oleifera* and safflower oil [107, 108]. The phytochemicals present in *M. oleifera* and safflower oil were obtained from the PubChem database, which offers chemical structures for docking purposes. Additionally, this study assessed the compound list using Lipinski’s rule of five and ADME properties to limit the number of compounds for docking. The software programs Hex 8.0.0 ( https://hex.loria.fr ) and FRED v3.2.0 ( https://www.eyesopen.com/oedocking ) were used for molecular docking. Hex 8.0.0 is an interactive program that calculates and displays feasible docking modes for proteins, using spherical polar Fourier correlations to accelerate the calculations. The FRED v3.2.0 software examines all possible protein-ligand poses in a systematic and non-stochastic manner. It applies filters for shape complementarity and chemical feature alignment before selection and optimization using the Chemgauss4 scoring function. Using binding affinities and ligand-protein interaction analysis, Khusro et al [107] identified five compounds as suitable inhibitors: 5-bis(1,1-dimethylethyl)-phenol, kaempferol, moringyne, niazimicin, and tetradecanoic acid. Nine compounds were selected from safflower oil using similar criteria [108]. These studies also lack the MD analysis of the selected ligands and *in vitro* assessment of the compounds.

A distinct methodology was employed by Cuccioloni et al [44] in their investigation of inhibitors targeting 8-hydroxy-5-deazaflavin:NADPH oxidoreductase (FNO), an enzyme present in methanogens. All the studies discussed above obtained the enzyme structures from X-ray crystallography results that were available in databases (i.e., RCSB database). However, the X-ray crystallography structure of FNO from *Methanobrevibacter smithii* used in this study was unavailable, so the researchers used the homology modeling method to obtain a predicted structure. The study used 8,012 ligands selected based on molecular weight and partition coefficient from the ZINC database. After molecular docking (using AutoDock Vina software), the researchers grouped the ligands into three groups according to their binding affinities and structural descriptors. Then, ten molecules representing the three groups were tested using the spectrofluorometric assay for FNO activity. The binding study was validated using a surface plasmon resonance (SPR) biosensor. SPR biosensors determined the kinetics of ligand-enzyme interactions, including the association and dissociation rates and the equilibrium dissociation constant [109]. According to this study, β-D-glucose pentaacetate, mangiferin, and baicalin exhibit promising inhibitory activities. Furthermore, the combination of docking studies with enzyme assays and binding studies provided a model process for validating the *in silico* work conducted in this study.

## CONCLUSION AND PERSPECTIVES

The scientific community and research organizations have recognized the importance of mitigating methane emissions from ruminant livestock. Although extensive research has been done, it is still necessary to find effective solutions to decrease methane emissions from livestock. While *in silico* drug development procedures have been used in human drug discovery programs, the application of *in silico* techniques to identify enzyme inhibitors specific to methanogens is a new approach in livestock-related research. Previous studies using *in silico* approaches have yielded significant results and provided new insights for reducing methane in the rumen. Notably, the discovery of 3-NOP as an inhibitor for MCR enzyme in methanogens has shown promising results in reducing methane under *in vitro* and *in vivo* conditions.

The *in silico* inhibitor discovery studies discussed in this paper offer possible methanogenesis inhibitors. However, most studies have not substantiated the effectiveness of the inhibitors *in vitro* or *in vivo*. Due to the complexity and variability of the rumen environment, the success of an inhibitor specifically targeting methanogens is limited. Therefore, results from *in silico* docking and dynamics studies should be confirmed using *in vitro* and *in vivo* experiments. Moreover, collaborative research that combines computational biology, medicinal chemistry, biochemistry, and animal sciences is of great importance for the success of these types of studies. The success of *in silico* research relies on the availability of high-resolution enzyme structures from methanogens. There is a need for more enzyme structures from rumen methanogen-specific enzymes, and future research should address this issue. Artificial intelligence (AI) could aid the search for rumen methanogenesis inhibitors. AI can analyze extensive amounts of data, recognize patterns that would be unfeasible for humans, and anticipate potential inhibitors’ efficacy, thus expediting the discovery process. Machine learning algorithms, a subset of AI, can be trained using existing data on known inhibitors and their effects on methanogens. These models can then predict the potential efficacy of new compounds. Furthermore, AI can assist in optimizing the chemical structure of these inhibitors to enhance their effectiveness and minimize potential side effects. However, there is a need for high-quality, annotated data to train AI models and model interpretability to understand the reasoning behind AI predictions. Validating the predictions made by AI in the laboratory can be a challenging and resource-intensive process. The AI models may need to fully capture the complexities of the interactions between enzymes and ligands. Ethical and regulatory considerations must be carefully addressed, including data privacy and the validation of AI-generated drugs.

## Figures and Tables

**Table 1:** Examples of ligand databases available for virtual screening of methanogenesis inhibitors

Database	Type of compounds	Website	Reference
PubChem	Pure, characterized chemical compounds and substances	https://pubchem.ncbi.nlm.nih.gov/	[68]
ChEMBL	Bioactive chemical molecules with drug-like properties	https://www.ebi.ac.uk/chembl/	[69]
ZINC20	Collection of commercially available compounds for virtual screening	https://zinc20.docking.org/	[70]
Enamine	Commercially available chemical compounds	https://enamine.net/compound-libraries	[71]
ChemDiv	Commercially available chemical compounds	https://www.chemdiv.com/	-
DrugBank	Drugs and drug targets	https://go.drugbank.com/	[72]
PSC-db	Plant secondary compounds	https://pscdb.appsbio.utalca.cl/viewSearch/index.php	[73]
SuperNatural 3	Compounds produced by living organisms	https://bioinf-applied.charite.de/supernatural_3/index.php	[74]
Seaweed Metabolites Database	Secondary metabolites of selected seaweeds	https://www.swmd.co.in/	[75]

**Table 2:** Example list of docking software and algorithms used for in silico docking^1)^

Docking software	Algorithm used	Reference
Autodock 4.2	Lamarckian genetic algorithm	[78]
AutoDock Vina	Lamarckian genetic algorithm	[79]
DOCK	Geometric algorithm	[80]
FRED	Non-stochastic method	[81]
GlamDock	Monte Carlo stochastic algorithm	[82]
GOLD	Genetic algorithm	[83]
ICM	Monte Carlo stochastic algorithm	[84]
ProPose	Incremental construction	[85]
Schrodinger’s Glide	Hierarchical method	[86]
Zdock	Fast fourier transform algorithm	[87]

1) The software list is given in alphabetical order without prioritizing their usability characteristics.

## References

[1] World Meteorological Organization (c2023). July 2023 is set to be the hottest month on record [Internet].

[2] Letcher TM, Letcher TM (2021). Global warming-a complex situation. Climate change: observed impacts on planet earth.

[3] Mehmood T, Hassan MA, Li X, Dervash MA, Wani AA (2023). Mechanism behind sources and sinks of major anthropogenic greeenhouse gasses. Climate change alleviation for sustainable progression.

[4] Ritchie H (c2020). Sector by sector: where do global greenhouse gas emissions come from? [Internet].

[5] United States Environmental Protection Agency (c2024). Understanding global warming potentials [Internet].

[6] International energy agency (c2023). Understanding methane emissions – global methane tracker 2023 [Internet].

[7] Arndt C, Hristov AN, Price WJ (2022). Full adoption of the most effective strategies to mitigate methane emissions by ruminants can help meet the 1.5 °C target by 2030 but not 2050. Proc Natl Acad Sci USA.

[8] Mackie RI, Kim H, Kim NK, Cann I (2024). Hydrogen production and hydrogen utilization in the rumen: key to mitigating enteric methane production. Anim Biosci.

[9] Beauchemin KA, Ungerfeld EM, Eckard RJ, Wang M (2020). Review: fifty years of research on rumen methanogenesis: lessons learned and future challenges for mitigation. Animal.

[10] Xie KL, Wang ZF, Guo YR, Zhang C, Zhu WH, Hou FJ (2022). Gentiana straminea supplementation improves feed intake, nitrogen and energy utilization, and methane emission of Simmental calves in northwest China. Anim Biosci.

[11] Uemoto Y, Tomaru T, Masuda M (2024). Exploring indicators of genetic selection using the sniffer method to reduce methane emissions from Holstein cows. Anim Biosci.

[12] Sinz S, Marquardt S, Soliva CR (2019). Phenolic plant extracts are additive in their effects against in vitro ruminal methane and ammonia formation. Asian-Australas J Anim Sci.

[13] Niu H, Xu Z, Yang HE, McAllister TA, Acharya S, Wang Y (2021). In vitro ruminal fermentation of fenugreek (Trigonella foenum-graecum L.) produced less methane than that of alfalfa (Medicago sativa). Anim Biosci.

[14] Dong L, Gao Y, Jing X (2022). Pretreatments of Broussonetia papyrifera: in vitro assessment on gas and methane production, fermentation characteristic, and methanogenic archaea profile. Anim Biosci.

[15] Henderson G, Cook GM, Ronimus RS (2018). Enzyme- and gene-based approaches for developing methanogen-specific compounds to control ruminant methane emissions: a review. Anim Prod Sci.

[16] Patra AK, Puchala R (2023). Methane mitigation in ruminants with structural analogues and other chemical compounds targeting archaeal methanogenesis pathways. Biotechnol Adv.

[17] Shinkai T, Takizawa S, Fujimori M, Mitsumori M (2024). The role of rumen microbiota in enteric methane mitigation for sustainable ruminant production. Anim Biosci.

[18] Patra A, Park T, Kim M, Yu Z (2017). Rumen methanogens and mitigation of methane emission by anti-methanogenic compounds and substances. J Anim Sci Biotechnol.

[19] Attwood GT, Altermann E, Kelly WJ, Leahy SC, Zhang L, Morrison M (2011). Exploring rumen methanogen genomes to identify targets for methane mitigation strategies. Anim Feed Sci Technol.

[20] Leahy SC, Kelly WJ, Altermann E (2013). The genome sequence of the rumen methanogen Methanobrevibacter ruminantium reveals new possibilities for controlling ruminant methane emissions. PLoS ONE.

[21] Shaker B, Ahmad S, Lee J, Jung C, Na D (2021). In silico methods and tools for drug discovery. Comput Biol Med.

[22] Adelusi TI, Oyedele AQK, Boyenle ID (2022). Molecular modeling in drug discovery. Inform Med Unlocked.

[23] Bender BJ, Gahbauer S, Luttens A (2021). A practical guide to large-scale docking. Nat Protoc.

[24] Chang Y, Hawkins BA, Du JJ, Groundwater PW, Hibbs DE, Lai F (2023). A guide to in silico drug design. Pharmaceutics.

[25] Cheng T, Li Q, Zhou Z, Wang Y, Bryant SH (2012). Structure-based virtual screening for drug discovery: a problem-centric review. AAPS J.

[26] Woese CR, Kandler O, Wheelis ML (1990). Towards a natural system of organisms: proposal for the domains archaea, bacteria, and eucarya. Proc Natl Acad Sci USA.

[27] Lambie SC, Kelly WJ, Leahy SC (2015). The complete genome sequence of the rumen methanogen Methanosarcina barkeri CM1. Stand Genomic Sci.

[28] Deppenmeier U (2002). The unique biochemistry of methanogenesis. Prog Nucleic Acid Res Mol Biol.

[29] Grabarse W, Mahlert F, Duin EC (2001). On the mechanism of biological methane formation: Structural evidence for conformational changes in methyl-coenzyme M reductase upon substrate binding. J Mol Biol.

[30] Chen H, Gan Q, Fan C (2020). Methyl-coenzyme M reductase and its post-translational modifications. Front Microbiol.

[31] Duin EC, Prakash D, Brungess C, Rosenzweig AC, Ragsdale SW (2011). Methyl-coenzyme M reductase from Methanothermobacter marburgensis.

[32] Berman HM, Westbrook J, Feng Z (2000). The protein data bank. Nucleic Acids Res.

[33] Duin EC, Wagner T, Shima S (2016). Mode of action uncovered for the specific reduction of methane emissions from ruminants by the small molecule 3-nitrooxypropanol. Proc Natl Acad Sci USA.

[34] Liu H, Wang J, Wang A, Chen J (2011). Chemical inhibitors of methanogenesis and putative applications. Appl Microbiol Biotechnol.

[35] Konisky J (1990). Inhibitory effects of 2-bromoethanesulfonate and protection by addition of coenzyme M in hydrogen-oxidizing marine enrichment cultures. FEMS Microbiol Ecol.

[36] Escalante-Semerena JC, Rinehart KL, Wolfe RS (1984). Tetrahydromethanopterin, a carbon carrier in methanogenesis. J Biol Chem.

[37] Karp PD, Billington R, Caspi R (2018). The BioCyc collection of microbial genomes and metabolic pathways. Brief Bioinform.

[38] Dumitru RV, Ragsdale SW (2004). Mechanism of 4-(β-D-ribofuranosyl)aminobenzene 5′-phosphate synthase, a key enzyme in the methanopterin biosynthetic pathway. J Biol Chem.

[39] Dumitru R, Palencia H, Schroeder SD (2003). Targeting methanopterin biosynthesis to inhibit methanogenesis. Appl Environ Microbiol.

[40] Miner JL, Ragsdale SW, Takacs JM, Board of Regents of the University of Nebraska (2003). Method for the inhibition of methanogenesis.

[41] Jumper J, Evans R, Pritzel A (2021). Highly accurate protein structure prediction with AlphaFold. Nature.

[42] Grinter R, Greening C (2021). Cofactor F420: an expanded view of its distribution, biosynthesis and roles in bacteria and archaea. FEMS Microbiol Rev.

[43] Joseph E, Le CQ, Nguyen T (2016). Evidence of negative cooperativity and half-site reactivity within an F420-dependent enzyme: kinetic analysis of F420H2:NADP+ oxidoreductase. Biochemistry.

[44] Cuccioloni M, Bonfili L, Cecarini V (2020). Structure/activity virtual screening and in vitro testing of small molecule inhibitors of 8-hydroxy-5-deazaflavin:NADPH oxidoreductase from gut methanogenic bacteria. Sci Rep.

[45] Warkentin E, Mamat B, Sordel-Klippert M (2001). Structures of F420H2:NADP+ oxidoreductase with and without its substrates bound. EMBO J.

[46] Neupane P, Bhuju S, Thapa N, Bhattarai HK (2019). ATP synthase: structure, function and inhibition. Biomol Concepts.

[47] Schäfer IB, Bailer SM, Düser MG (2006). Crystal structure of the archaeal A1AO ATP synthase subunit B from Methanosarcina mazei Gö1: implications of nucleotide-binding differences in the major A1AO subunits A and B. J Mol Biol.

[48] Aung HL, Dey D, Janssen PH, Ronimus RS, Cook GM (2015). A high-throughput screening assay for identification of inhibitors of the A1AO-ATP synthase of the rumen methanogen Methanobrevibacter ruminantium M1. J Microbiol Methods.

[49] Vonck J, Pisa KY, Morgner N, Brutschy B, Müller V (2009). Three-dimensional structure of A1A0 ATP synthase from the hyperthermophilic archaeon Pyrococcus furiosus by electron microscopy. J Biol Chem.

[50] Kumar A, Manimekalai MSS, Grüber G (2008). Structure of the nucleotide-binding subunit B of the energy producer A 1A0 ATP synthase in complex with adenosine diphosphate. Acta Crystallogr D Struct Biol.

[51] Řezanka T, Kyselová L, Murphy DJ (2023). Archaeal lipids. Prog Lipid Res.

[52] Johnson BP, Kumar V, Scull EM, Thomas LM, Bourne CR, Singh S (2022). Molecular basis for the substrate promiscuity of isopentenyl phosphate kinase from Candidatus methanomethylophilus alvus. ACS Chem Biol.

[53] Miller TL, Wolin MJ (2001). Inhibition of growth of methane-producing bacteria of the ruminant forestomach by hydroxymethylglutaryl~SCoA reductase inhibitors. J Dairy Sci.

[54] Guerra B, Recio C, Aranda-Tavío H (2021). The mevalonate pathway, a metabolic target in cancer therapy. Front Oncol.

[55] Ábrego-Gacía A, Poggi-Varaldo HM, Robles-González V (2021). Lovastatin as a supplement to mitigate rumen methanogenesis: an overview. J Anim Sci Biotechnol.

[56] Sneha P, Doss CGP, Donev R (2016). Molecular dynamics: new frontier in personalized medicine. Advances in protein chemistry and structural biology.

[57] Grochowski LL, Xu H, White RH (2006). Methanocaldococcus jannaschii uses a modified mevalonate pathway for biosynthesis of isopentenyl diphosphate. J Bacteriol.

[58] Mabanglo MF, Schubert HL, Chen M, Hill CP, Poulter CD (2010). X-ray structures of isopentenyl phosphate kinase. ACS Chem Biol.

[59] National Library of Medicine (2023). isopentenyl phosphate kinase [Methanobrevibacter ruminantium] [Internet]. Protein.

[60] Ekins S, Mestres J, Testa B (2007). In silico pharmacology for drug discovery: methods for virtual ligand screening and profiling. Br J Pharmacol.

[61] Kontoyianni M, Lazar IM, Kontoyianni M, Lazar AC (2018). Docking and virtual screening in drug discovery. Proteomics for Drug Discovery Methods in Molecular Biology.

[62] Kumar V, Krishna S, Siddiqi MI (2015). Virtual screening strategies: recent advances in the identification and design of anti-cancer agents. Methods.

[63] Stoll F, Göller AH, Hillisch A (2011). Utility of protein structures in overcoming ADMET-related issues of drug-like compounds. Drug Discov Today.

[64] Fährrolfes R, Bietz S, Flachsenberg F (2017). Proteins plus: a web portal for structure analysis of macromolecules. Nucleic Acids Res.

[65] Muhammed MT, Aki-Yalcin E (2019). Homology modeling in drug discovery: overview, current applications, and future perspectives. Chem Biol Drug Des.

[66] Guterres H, Park SJ, Jiang W, Im W (2021). Ligand-binding-site refinement to generate reliable holo protein structure conformations from apo structures. J Chem Inf Model.

[67] Villar HO, Cavasotto CN (2015). Library design, chemical space, and drug likeness. Silico Drug Discovery and Design.

[68] Kim S, Chen J, Cheng T (2023). PubChem 2023 update. Nucleic Acids Res.

[69] Mendez D, Gaulton A, Bento AP (2019). ChEMBL: towards direct deposition of bioassay data. Nucleic Acids Res.

[70] Irwin JJ, Tang KG, Young J (2020). ZINC20 - A free ultralarge-scale chemical database for ligand discovery. J Chem Inf Model.

[71] Grygorenko OO (2021). Enamine ltd.: the science and business of organic chemistry and beyond. European J Org Chem.

[72] Wishart DS, Knox C, Guo AC (2006). DrugBank: a comprehensive resource for in silico drug discovery and exploration. Nucleic Acids Res.

[73] Valdés-Jiménez A, Peña-Varas C, Borrego-Muñoz P (2021). Psc-db: a structured and searchable 3d-database for plant secondary compounds. Molecules.

[74] Gallo K, Kemmler E, Goede A (2023). SuperNatural 3.0 - a database of natural products and natural product-based derivatives. Nucleic Acids Res.

[75] Davis GDJ, Vasanthi AHR (2011). Seaweed metabolite database (SWMD): a database of natural compounds from marine algae. Bioinformation.

[76] Sorokina M, Steinbeck C (2020). Review on natural products databases: where to find data in 2020. J Cheminform.

[77] Agu PC, Afiukwa CA, Orji OU (2023). Molecular docking as a tool for the discovery of molecular targets of nutraceuticals in diseases management. Sci Rep.

[78] Morris GM, Ruth H, Lindstrom W (2009). AutoDock4 and AutoDockTools4: automated docking with selective receptor flexibility. J Comput Chem.

[79] Eberhardt J, Santos-Martins D, Tillack AF, Forli S (2021). AutoDock Vina 1.2.0: new docking methods, expanded force field, and python bindings. J Chem Inf Model.

[80] Coleman RG, Carchia M, Sterling T, Irwin JJ, Shoichet BK (2013). Ligand pose and orientational sampling in molecular docking. PLoS ONE.

[81] McGann M (2011). FRED pose prediction and virtual screening accuracy. J Chem Inf Model.

[82] Tietze S, Apostolakis J (2007). GlamDock: development and validation of a new docking tool on several thousand protein-ligand complexes. J Chem Inf Model.

[83] Verdonk ML, Cole JC, Hartshorn MJ, Murray CW, Taylor RD (2003). Improved protein-ligand docking using GOLD. Proteins.

[84] Neves MAC, Totrov M, Abagyan R (2012). Docking and scoring with ICM: the benchmarking results and strategies for improvement. J Comput Aided Mol Des.

[85] Hogues H, Gaudreault F, Corbeil CR, Deprez C, Sulea T, Purisima EO (2018). ProPOSE: direct exhaustive protein-protein docking with side chain flexibility. J Chem Theory Comput.

[86] Friesner RA, Banks JL, Murphy RB (2004). Glide: a new approach for rapid, accurate docking and scoring. 1. method and assessment of docking accuracy. J Med Chem.

[87] Pierce BG, Wiehe K, Hwang H, Kim BH, Vreven T, Weng Z (2014). ZDOCK server: interactive docking prediction of protein-protein complexes and symmetric multimers. Bioinformatics.

[88] Lill M, Kortagere S (2013). Virtual screening in drug design. In Silico Models for Drug Discovery Methods in Molecular Biology.

[89] Herrera-Acevedo C, Perdomo-Madrigal C, de Luis JAS, Scotti L, Scotti MT, Scotti MT, Bellera CL (2022). Drug discovery paradigms: target-based drug discovery. Drug target selection and validation.

[90] Trott O (c2018). Vina video tutorial [Internet].

[91] Huey R, Morris GM (c2003). Using AutoDock with AutoDockTools: A Tutorial [Internet]. AutoDockTools.

[92] Karplus M, McCammon JA (2002). Molecular dynamics simulations of biomolecules. Nat Struct Biol.

[93] Abraham MJ, Murtola T, Schulz R (2015). Gromacs: high performance molecular simulations through multi-level parallelism from laptops to supercomputers. SoftwareX.

[94] Salomon-Ferrer R, Case DA, Walker RC (2013). An overview of the Amber biomolecular simulation package. Wiley Interdiscip Rev Comput Mol Sci.

[95] Phillips JC, Hardy DJ, Maia JDC (2020). Scalable molecular dynamics on CPU and GPU architectures with NAMD. J Chem Phys.

[96] Brooks BR, Brooks CL, Mackerell AD (2009). CHARMM: the biomolecular simulation program. J Comput Chem.

[97] Bowers KJ, Chow DE, Xu H (2006). Scalable algorithms for molecular dynamics simulations on commodity clusters.

[98] De Vivo M, Masetti M, Bottegoni G, Cavalli A (2016). Role of molecular dynamics and related methods in drug discovery. J Med Chem.

[99] Paul F, Thomas T, Roux B (2020). Diversity of long-lived intermediates along the binding pathway of imatinib to Abl kinase revealed by MD simulations. J Chem Theory Comput.

[100] Aier I, Varadwaj PK, Raj U (2016). Structural insights into conformational stability of both wild-type and mutant EZH2 receptor. Sci Rep.

[101] Schrodinger Inc (c2021). Simulation interactions diagram report [Internet].

[102] Sharma J, Kumar Bhardwaj V, Singh R, Rajendran V, Purohit R, Kumar S (2021). An in-silico evaluation of different bioactive molecules of tea for their inhibition potency against non structural protein-15 of SARS-CoV-2. Food Chem.

[103] Arokiyaraj S, Stalin A, Shin H (2019). Anti-methanogenic effect of rhubarb (Rheum spp.) – an in silico docking studies on methyl-coenzyme M reductase (MCR). Saudi J Biol Sci.

[104] Kim KH, Arokiyaraj S, Lee J (2016). Effect of rhubarb (Rheum spp.) root on in vitro and in vivo ruminal methane production and a bacterial community analysis based on 16S rRNA sequence. Anim Prod Sci.

[105] Bulusu G, Desiraju GR (2020). Strong and weak hydrogen bonds in protein–ligand recognition. J Indian Inst Sci.

[106] Dinakarkumar Y, Rajabathar JR, Arokiyaraj S (2021). Anti-methanogenic effect of phytochemicals on methyl-coenzyme M reductase—potential: in silico and molecular docking studies for environmental protection. Micromachines.

[107] Khusro A, Aarti C, Salem AZM, Pliego AB, Rivas-Caceres RR (2020). Methyl-coenzyme M reductase (MCR) receptor as potential drug target for inhibiting methanogenesis in horses using Moringa oleifera L.: an in silico docking study. J Equine Vet Sci.

[108] Khusro A, Sahibzada MUK, Khan SU (2022). Anti-methanogenic traits of safflower oil compounds against methyl-coenzyme m reductase receptor in equines: an in silico docking analysis. J Equine Vet Sci.

[109] Murali S, Rustandi RR, Zheng X, Payne A, Shang L (2022). Applications of surface plasmon resonance and biolayer interferometry for virus–ligand binding. Viruses.

